# Targeting the Hsp90-Cdc37-client protein interaction to disrupt Hsp90 chaperone machinery

**DOI:** 10.1186/s13045-018-0602-8

**Published:** 2018-04-27

**Authors:** Ting Li, Hu-Lin Jiang, Yun-Guang Tong, Jin-Jian Lu

**Affiliations:** 1 State Key Laboratory of Quality Research in Chinese Medicine, Institute of Chinese Medical Sciences, University of Macau, Avenida da Universidade, Taipa, Macau China; 2 State Key Laboratory of Natural Medicines, China Pharmaceutical University, Nanjing, China; 3 Department of Pathology, Xinxiang Medical University, 601 East Jinsui Ave, Xinxiang, Henan China; 4 Omigen, Inc., 15375 Barranca Pkwy, Irvine, CA H106 USA

**Keywords:** Hsp90 chaperone machinery, Cdc37, Kinase client, Protein interaction

## Abstract

Heat shock protein 90 (Hsp90) is a critical molecular chaperone protein that regulates the folding, maturation, and stability of a wide variety of proteins. In recent years, the development of Hsp90-directed inhibitors has grown rapidly, and many of these inhibitors have entered clinical trials. In parallel, the functional dissection of the Hsp90 chaperone machinery has highlighted the activity disruption of Hsp90 co-chaperone as a potential target. With the roles of Hsp90 co-chaperones being elucidated, cell division cycle 37 (Cdc37), a ubiquitous co-chaperone of Hsp90 that directs the selective client proteins into the Hsp90 chaperone cycle, shows great promise. Moreover, the Hsp90-Cdc37-client interaction contributes to the regulation of cellular response and cellular growth and is more essential to tumor tissues than normal tissues. Herein, we discuss the current understanding of the clients of Hsp90-Cdc37, the interaction of Hsp90-Cdc37-client protein, and the therapeutic possibilities of targeting Hsp90-Cdc37-client protein interaction as a strategy to inhibit Hsp90 chaperone machinery to present new insights on alternative ways of inhibiting Hsp90 chaperone machinery.

**Electronic supplementary material:**

The online version of this article (10.1186/s13045-018-0602-8) contains supplementary material, which is available to authorized users.

## Background

Heat shock protein 90 (Hsp90) is a critically conserved protein and one of the major molecular chaperones within eukaryotic cells [1]. It is the key component of the Hsp90 chaperone machinery, which facilitates protein folding and regulates the stabilization and activity of a large number of client proteins [2–4]. Hsp90 client proteins originate from distinct functional classes, including transcription factors (e.g., HIF1α, ATF3, and p53), steroid hormone receptors (e.g., estrogen receptor, glucocorticoid receptor, and progesterone receptor), and kinases (e.g., EGFR, B-raf, and SRC). Many of these client proteins are commonly overexpressed and/or frequently mutated in cancer cells [5–7]. Therefore, targeting Hsp90 offers a promising multi-therapeutic strategy for cancer treatment.

Hsp90 family contains five subfamilies, which include cytosolic HSP90A, endoplasmic reticulum-localized HSP90B, chloroplast HSP90C, mitochondrial TNFR-associated protein, and bacterial high-temperature protein G [2, 8]. In this review, we use the term Hsp90 to refer to these Hsp90 members when we do not explicitly distinguish the differences between them. Hsp90 functions as a homodimer, and each Hsp90 monomer comprises three critical conserved domains. The amino-terminal domain that is also referred to as N-terminal domain mediates its ATP binding activity, the middle domain is the center of ATP hydrolysis and client binding, and the carboxy-terminal domain that is also referred to as the C-terminal domain is responsible for its dimerization [2, 9, 10]. ATP binding results in the rearrangement of the three domains, which induces a shift from an open conformation to a closed conformation [11]. The ATPase activity of Hsp90 is thought to be essential for the chaperone cycle of Hsp90 chaperone machinery [2]. Hsp90-directed agents that block ATP binding to Hsp90 have been established, such as geldanamycin (GM)-based inhibitors (GM, 17-AAG, 17-DMAG, and IPI-504), purine-based inhibitors (PU3, BIIBO21, PU-H71, MPC-3100, and CUDC-305), and radicicol-based inhibitors (NVP-AUY922, STA-9090, AT-13387, and KW-2478) [12–17]. Preclinical data have suggested Hsp90 as a potential anticancer target; however, the clinical trials have been halted or postponed in many cases. So far, none of the inhibitors for Hsp90 intervention have been approved for clinical application, which may be due to toxicity and heat shock response upon clinical treatment [2, 18, 19]. As a consequence, the strategy of blocking Hsp90’s ATP-binding activity for the development of Hsp90 inhibitor is still under controversy.

Alongside the Hsp90 chaperone, co-chaperones are crucial regulators that drives the diverse functions of Hsp90 chaperone machinery. These co-chaperones, such as Hsp70/Hsp90 organizing protein (HOP), cell division cycle 37 (Cdc37), small glutamine-rich TPR-containing protein alpha (SGTA), TPR repeat-containing protein associated with Hsp90 (TAH1), activator of Hsp90 ATPase protein 1 (AHA1), protein phosphatase 5 (PP5), FK506-binding protein 5 (FKBP5), carboxyl terminus of HSC70-interacting protein (CHIP), cyclophilin 40 (CYP40), p23, tetratricopeptide repeat domain 4 (TTC4), and uncoordinated mutant number 45 (Unc45), in concert with Hsp90 to form transient complexes not only mediate the Hsp90 ATPase cycle but also direct a broad range of specific clients to Hsp90 [2]. Among them, Cdc37, also known as p50, is one of the best-studied co-chaperones of Hsp90 [20–23] and proposed to be exclusively associated with protein kinases [24, 25]. Recent progress has allowed the interactions of Hsp90-Cdc37-client protein to be defined [20, 26, 27]. Herein, we discuss the current understanding of the clients of Hsp90-Cdc37, the interaction of Hsp90-Cdc37-client, and the prospects of targeting Hsp90-Cdc37-client interaction as a target for therapeutic intervention of the Hsp90 chaperone machinery.

### The client proteins of Hsp90-Cdc37

**Fig. 1:**
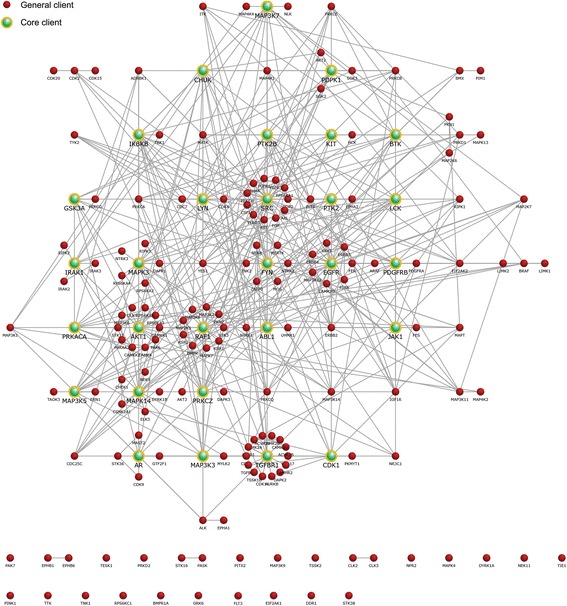
The protein–protein interaction of Hsp90-Cdc37’s client proteins. Using the FunRich tool to analyze the protein–protein interaction of Hsp90-Cdc37’s client proteins. 449 PPI pairs are formed by these 267 proteins in total. PPI: protein–protein interaction

Cdc37, first identified as a cell cycle protein in 1980 [28], was found to be an important intracellular cofactor of Hsp90 in 1981 [25, 29]. As a specific co-chaperone, Cdc37 is a selectively acquired recruiter for Hsp90 chaperone machinery to control the entry of diverse protein kinases [30]. The majority of kinase proteins interact with both Hsp90 and its co-chaperone Cdc37, and the Hsp90-mediated maturation of kinases is proposed to be strictly dependent on Cdc37 [20, 26]. To date, nearly 300 client proteins of Hsp90-Cdc37, which include diverse proteins such as EGFR, SRC, B-raf, AKT1, and CDK4, have been found [20, 31]. To obtain a better understanding of the biological function and molecular interaction of the client proteins of Hsp90-Cdc37, a stand-alone software tool FunRich (version 3.0), which is mainly used for the functional enrichment and interaction network analysis, was applied [32]. A well-annotated list of Hsp90-Cdc37 client proteins was maintained and updated by the Picard laboratory, a research group that focus on the development of Hsp90 (see the online link: https://www.picard.ch/downloads/Cdc37interactors.pdf) [31], and 267 proteins were successfully matched with the FunRich database (human only). Overall, our results demonstrated that the molecular functions of these proteins were mainly implicated in regulation of protein serine/threonine kinase activity (143 proteins), and the biological processes in which they mainly participated were cell communication (192 proteins) and signal transduction (214 proteins) (detailed data are shown in Additional file 1). These client proteins are involved in various biological pathways, such as the glypican pathway, IFN-γ pathway, TRAIL signaling pathway, ERBB receptor signaling pathway, and VEGF/VEGFR signaling network. Actually, most of the proteins do not operate alone but interact with each other and work in complexes while performing their functions in cellular progresses. The protein–protein interaction (PPI) plays a critical role in the signal transduction pathways and networks during diverse physiological processes [33]. In the present study, the PPI network visualization and its analysis were also performed, and 449 PPI pairs were formed by the 267 different client proteins (Fig. 1a). Among them, 29 client proteins, including SRC, EGFR, FYN, Raf-1, and AKT1, formed at least 10 pairs in the PPI network and were defined as core client proteins (Table 1). Different EGFR family members with various biological activities could form the ligand-triggered heterodimers with family members, resulting in activation of complex signals [34–36]. IGF1R was also identified to form heterodimers with insulin receptor (gene symbol: IRSNN) and EGFR family members, conferring resistance to homo-therapy [37, 38]. The Raf-1/B-raf-formed heterodimers exhibit highly increased kinase activity than the respective homodimers [39]. Clearly, an individual protein alone cannot be a simple predictor of drug efficiency; functional protein–protein dimers with distinct pharmacological properties are also required [40–44]. Herein, the complex protein network and sundry PPI pairs formed by these Hsp90-Cdc37 clients in turn heightened the understanding of Cdc37 as a key factor in Hsp90 chaperone machinery.

**Table 1:** The core client proteins. The 29 client proteins formed at least 10 pairs in the PPI network

Item	Protein name	Gene symbol	Molecular functions	Partner of PPI pair
1	SRC	SRC	Kinase, tyrosine-protein kinase, transferase	MAP4K1, PRKCA, PRKCE, RET, TYRO3, PTK2B, CHUK, DDR2, ADRBK1, EGFR, BMX, KIT, PDGFRB, RAF1, ERBB2, IKBKB, MATK, PRKCZ, ABL1, PTK2, AXL, AR, PRKACA, CDC25C, AKT1, ITK, FYN, IGF1R, CSF1R, PRKCH, PDPK1, EPHA4, LYN, PRKD1, FGR
2	EGFR	EGFR	Developmental protein, host cell receptor for virus entry, kinase, receptor, tyrosine-protein kinase, transferase	MAP4K1, EPHA2, AR, LYN, PDGFRB, PRKD1, FER, SRC, PTK2B, ERBB3, MAP3K12, CDK1, YES1, ERBB2, PRKCA, IGF1R, PTK2, RIPK1, MAP3K14, CAMK2A, ERBB4, VAV3, PTK6, ADRBK1, TNK2, PRKACA, CAMK2G, MAPK14
3	FYN	FYN	Developmental protein, kinase, tyrosine-protein kinase, transferase	ITK, NTRK2, RAF1, NR3C1, ZAP70, TNK2, PRKCQ, PRKCE, TYK2, PTK2B, MAP4K1, CSF1R, TYRO3, PTK2, EPHA4, SRC, CDK1, YES1, PDGFRB, ABL1, PRKCZ, MAPT, NEK8, KIT, BTK, MOS, PRKCH
4	Raf-1	RAF1	Kinase, serine/threonine-protein kinase, transferase	MAP3K1, PRKCZ, FYN, PDGFRB, AR, STK3, BRAF, SRC, KSR1, AKT1, PRKCA, JAK1, MAPK3, PRKCE, MAPK7, DMPK, PRKACA, MAP3K5, NR3C1, LCK, KSR2
5	AKT1	AKT1	Kinase, serine/threonine-protein kinase, transferase	PDPK1, PRKCQ, PKN2, AR, CHUK, MAP3K8, RAF1, BRAF, MAP3K11, PRKCZ, IKBKB, SRC, MAPK14, CAMKK1, AKT2, IRAK1, PAK6, MAP3K5, GSK3A, CHEK1
6	ERK1	MAPK3	Kinase, serine/threonine-protein kinase, transferase	DAPK1, RPS6KA2, RPS6KA3, RPS6KA4, MAPK14, PRKCZ, RPS6KA1, KSR2, PRKCE, LCK, RAF1, CDC25C, MAP3K14, NTRK1, LYN, NTRK3, ADRBK1, BRAF, RET
7	P38	MAPK14	Kinase, serine/threonine-protein kinase, transferase	IRAK1, DYRK1B, RPS6KA4, RPS6KA3, MAPK3, MAP3K7, CSNK2A1, TGFBR1, MAP2K6, MAP2K7, CAMKK2, AKT1, RPS6KA5, CDC25C, PKN1, ELK3, RET, EGFR
8	FAK1	PTK2	Kinase, tyrosine-protein kinase, transferase	EPHA2, LYN, BMX, RET, IGF1R, SRC, EGFR, FLT4, FYN, FGR, PDGFRB, ERBB2, YES1, LCK, PTK2B, ERBB3, RIPK1
9	IKK-β	IKBKB	Kinase, serine/threonine-protein kinase, transferase	CHUK, MAP3K7, EIF2AK2, MAP3K14, MAP3K1, PRKCQ, PRKCB, MAP3K3, PRKCZ, SRC, TGFBR1, MAP3K11, AKT1, ACVR1, IRAK1, TBK1
10	TGFR-1	TGFBR1	Kinase, receptor, serine/threonine-protein kinase, transferase	CHUK, CDK17, AMHR2, DAPK2, AURKB, TGFBR2, MAPK14, CDK14, CDK6, IKBKB, MAP3K7, TSSK1B, NEK8, ITK, ACVR1, CDK4
11	LYN	LYN	Kinase, tyrosine-protein kinase, transferase	TYK2, PTK2, MATK, EGFR, KIT, CDK2, CDK1, BTK, CDK4, CSF1R, PTK2B, PRKCQ, MAPK3, MAP4K1, SRC, MAP3K3
12	PKC2	PRKCZ	Kinase, serine/threonine-protein kinase, transferase	RAF1, DAPK3, MAPK3, JAK1, PDPK1, IKBKB, SRC, PRKCQ, AKT3, AKT1, GSK3A, MAP2K5, IRAK1, BTK, FYN, PRKCA
13	PDK1	PDPK1	Activator, kinase, serine/threonine-protein kinase, transferase	PKN1, AKT1, PRKACA, PKN2, RPS6KA1, RPS6KA3, PRKCZ, PTK2B, PRKCE, SGK3, AKT2, PRKCB, SRC, SGK2, AKT3
14	IKK-α	CHUK	Kinase, serine/threonine-protein kinase, transferase	MAP3K8, MAP3K7, IKBKB, TGFBR1, SRC, MAP3K14, AKT1, AKT2, IRAK1, PRKCB, EIF2AK2, MAP3K11, PRKCQ, CHEK1, MAP3K3
15	MEKK7	MAP3K7	Kinase, serine/threonine-protein kinase, transferase	CHUK, IKBKB, MAP4K1, MAP2K7, MAPK14, MAP2K6, MAP3K5, RIPK1, NLK, TGFBR1, MAP3K14, MAP3K3, EIF2AK2, MAP4K4, IRAK1
16	BTK	BTK	Kinase, tyrosine-protein kinase, transferase	PRKCQ, PRKD1, LYN, JAK1, ABL1, BMX, IRAK1, PRKCE, PRKCZ, PRKCB, HCK, FYN, KIT, ITK, PRKCA
17	ABL	ABL1	DNA-binding, kinase, tyrosine-protein kinase, transferase	HCK, MAP4K1, PRKD1, SRC, NTRK1, NEK8, KIT, BTK, JAK1, ZAP70, CDK1, FYN, MAPT, UHMK1
18	MEKK5	MAP3K5	Kinase, serine/threonine-protein kinase, transferase	MAP2K7, EIF2AK2, MAP3K6, MAP3K7, MAP2K6, MAP3K2, MAP3K3, PRKAA2, RAF1, ERN1, AKT1, IGF1R, ALK
19	LCK	LCK	Kinase, tyrosine-protein kinase, transferase	PRKCA, ZAP70, ITK, PRKCQ, MAPK3, KIT, CDC25C, PRKACA, PTK2B, AXL, PTK2, RAF1, NR3C1
20	FAK2	PTK2B	Kinase, tyrosine-protein kinase, transferase	SRC, ZAP70, EGFR, FYN, PDPK1, ERBB3, JAK1, LYN, MATK, LCK, ERBB2, PTK2, FGFR3
21	PKACA	PRKACA	Kinase, serine/threonine-protein kinase, transferase	PDPK1, STK11, NR3C1, SRC, GSK3A, CAMKK2, LCK, ADRBK1, RAF1, MAP3K3, EGFR, BRAF
22	IRAK	IRAK1	Kinase, serine/threonine-protein kinase, transferase	MAPK14, CHUK, IRAK3, IRAK2, PRKCZ, MAP3K7, BTK, AKT1, RIPK2, CAMKK2, IKBKB, NTRK3
23	JAK1	JAK1	Kinase, tyrosine-protein kinase, transferase	IGF1R, PRKCZ, TYK2, RAF1, PTK2B, EIF2AK2, BTK, ABL1, PDGFRA, FES, PDGFRB, FER
24	CDK1	CDK1	Host cell receptor for virus entry, kinase, receptor, serine/threonine-protein kinase, transferase	PKMYT1, CDC25C, LYN, EGFR, CSNK2A1, AR, PRKCB, MAPT, FYN, ABL1, AURKB, TGFBR2
25	PDGFR-1	PDGFRB	Developmental protein, kinase, receptor, tyrosine-protein kinase, transferase	VAV3, RAF1, EGFR, SRC, PDGFRA, TYK2, ARAF, FYN, YES1, EIF2AK2, JAK1, PTK2
26	MEKK3	MAP3K3	Kinase, serine/threonine-protein kinase, transferase	MAP3K2, ALK, MAP2K5, IKBKB, MAP3K7, MYLK2, MAP3K5, PRKACA, RIPK1, CHUK, LYN
27	CD117	KIT	Kinase, receptor, tyrosine-protein kinase, transferase	LYN, SRC, MATK, HCK, YES1, LCK, ABL1, FYN, BTK, PRKCA, PRKCB
28	AR	AR	Activator, DNA-binding, receptor	PAK6, AKT1, EGFR, GTF2F1, RAF1, GSK3A, NR3C1, CDK6, SRC, CDK1, CDK9
29	GSK3α	GSK3A	Kinase, serine/threonine-protein kinase, signal transduction inhibitor, transferase	PRKCA, AR, MAPT, PRKACA, PRKCZ, SGK3, MAP3K11, PRKCB, AKT1, PRKCG

### The interaction of Hsp90-Cdc37-client protein

**Fig. 2:**
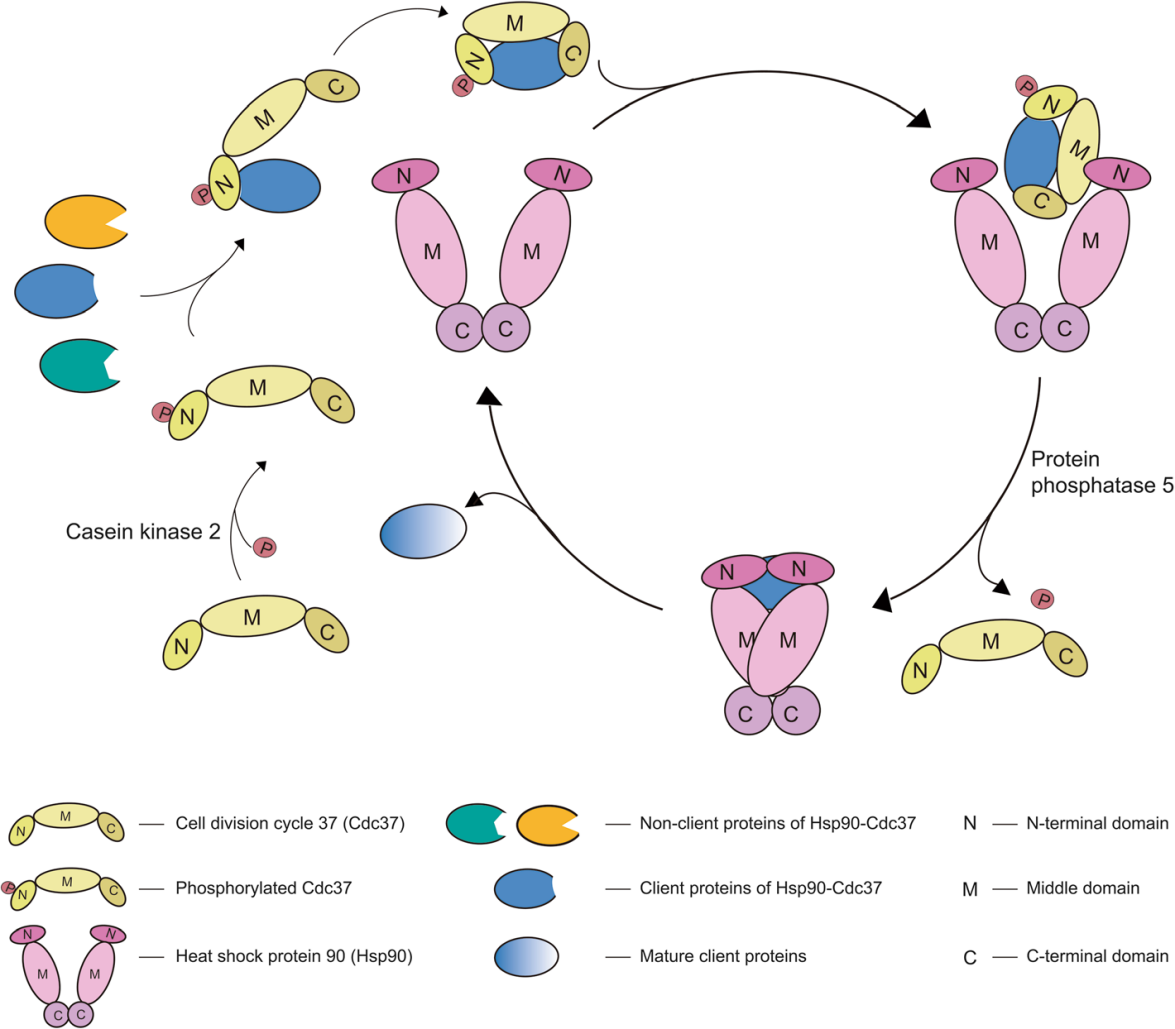
A speculative model for the Hsp90-Cdc37-client protein cycle. Cdc37 first tests the proper substrates and establishes stable connection with the client protein to create a Cdc37-client protein binary complex. Then, the binary complex binds to Hsp90 to form a ternary complex. The formation of the Hsp90-Cdc37-client protein ternary complex finally facilitates client protein loading onto the Hsp90 chaperone machinery. It is worthy of note that Cdc37 will be phosphorylated by casein kinase 2 (CK2) at Ser13 before connecting with client proteins and dephosphorylated by the protein phosphatase 5 (PP5) before client protein release

Various studies in recent years have helped determine how Cdc37 enables Hsp90 chaperone machinery to recognize specific client proteins progressively; however, the full-length structures of client protein with either Hsp90 or Cdc37 remain to be elucidated [1, 2, 27, 45]. Similar to Hsp90, Cdc37 contains three domains (N-terminal domain, M-domain, and the C-terminal domain) that function cooperatively [26, 27]. Cdc37 acts as a structural defect discriminator and a kinase sorting module in the Hsp90 chaperone machinery [46]. As shown in Fig. 2, Cdc37 first tests the proper substrates and establishes stable connection with the client protein to create a Cdc37-client protein binary complex. Then, the binary complex binds to Hsp90 to form a ternary complex. The formation of the Hsp90-Cdc37-client protein ternary complex finally facilitates client protein loading onto the Hsp90 chaperone machinery.

Of note, Cdc37 will be phosphorylated by casein kinase 2 (CK2) at Ser13 before connecting with client proteins [47] and dephosphorylated by the protein phosphatase PP5 before client protein release [48]. Proper phosphoserine of Cdc37 at Ser13 is necessary for Hsp90 chaperone machinery to recognize the structural perturbation at the client protein domain and helps Cdc37 to stabilize its own structure and participate in the interaction with Hsp90 [27]. These works have heightened the understanding of the phosphorylation on Cdc37’s conformation and ability to bind to Hsp90. Besides phospho-Ser13 of Cdc37, previous studies have recognized GSGSFG, a glycine-rich motif, as a unique sequence that is necessary for the stable client protein’s interaction with Cdc37 [49, 50], and the adjoining loop linking the α-C-helix to the β4-strand and the α-C-helix of the client protein also appears to be the primary determinant to be recognized by Cdc37 [51]. The difference of C-terminal portions within client proteins takes part in determining the sharp affinity activity with Cdc37, which may explain the different affinities between CDK4 (Cdc37-dependent protein) and CDK2 (non-Cdc37-dependent protein) despite the similar glycine-rich loop in the N-terminal portions [49]. Nevertheless, based on the analysis using FunRich tool, more than 50% (*P* < 0.001) of the client proteins contain the serine/threonine kinase catalytic domain (S-TKc), suggesting that this kind of domain may be preferable than others. Even though the definitive features of Cdc37 and client proteins have not yet been clearly illustrated, several key portions are crucial for the physical association of client protein and Cdc37 and determine the triage process regarding Cdc37’s dependence.

As for the binding site of Cdc37 and Hsp90, the classic one in humans is the middle domain of Cdc37 bond to the N-terminal domain of Hsp90 (shown in Fig. 2). Cdc37 uses the N-terminal domain to test the proper substrates and applies its C-terminal region to build the stable connection with the client protein. The Cdc37-client protein complex will then bind to the N-terminal domain of Hsp90 via the M-domain of Cdc37 [1, 2, 27, 45]. Specifically, the residues 127–163 within the middle domain of Cdc37 were recognized as the inter-domain switch to sense the proper conformation of Hsp90 and regulate the binding activity of Cdc37-client protein complex with Hsp90 [52]. In addition, a second interaction site of Hsp90 for Cdc37 within *Caenorhabditis elegans* (CeCdc37) was uncovered recently, which is regulated by the binding of N-terminal domain of CeCdc37 to the middle domain of Hsp90 [30, 53]. These two interactions utilized by Cdc37 within different species seem to function relevantly and mediate the conformational change and the ATPase activity of Hsp90.

Most recently, the client protein within the Hsp90-Cdc37-client protein ternary complex was found to bind to the separated sides of Hsp90 using its two elongated and non-native lobes [27]. This structure suggested that the client protein remains in an uncompleted folded status even after the conformation of the Hsp90-Cdc37-client protein ternary complex, and it still relies on the subsequent function of Hsp90 to reach maturity. This finding provides further evidence for the heavy dependence of client protein maturation on the cooperation of Cdc37 and Hsp90.

### Targeting Hsp90-Cdc37-client protein interaction to block Hsp90 chaperone machinery

As the majority of kinases are adversely affected by Hsp90-Cdc37, drug design targeting Hsp90-Cdc37-client protein interaction has been highlighted as a promising novel strategy. Investigating technically feasible methods to modulate Hsp90-Cdc37 activity is of considerable importance. Based on the nature of Hsp90-Cdc37-client protein interaction, there are three potential categories that are likely to disrupt the function of Hsp90 chaperone machinery: targeting Cdc37, Cdc37-client protein interaction, and Hsp90-Cdc37 interaction.

#### Targeting Cdc37

Cdc37 has an increased level in proliferating tissues and organs and is highly expressed in certain tumors, such as prostate cancer [54]. Therefore, the tumor cells, rather than normal cells, have increasing dependency on the Cdc37 level. Moreover, as the recognizer of client protein being tied to the Hsp90 chaperone system, Cdc37 is primarily and specifically interacted with the kinase protein, whereas Hsp90 is widely associated with many classes of client proteins (transcription factors, steroid hormone receptors, and kinases) [55]. The absence of Cdc37 can only disrupt the interactions with kinase clients but not the interactions with non-kinase clients [20, 56]. Cdc37 is needed for both maturation and activation of client proteins, which may be dependent on the differences of species and tissue. Silencing Cdc37 using shRNA disrupts the Hsp90 chaperone machinery via impairing the association of client protein with Hsp90 and prevents protein maturation, which may subsequently induce a proteasomal degradation of client proteins, and finally suppresses the proliferation of human colon cancer cells [56]. Additionally, depletion of Cdc37 could also block the activities of many client proteins, instead of global degradation of client proteins. This blockage resulted in the suppression of multiple pathways (e.g., MAPKs and androgen-induced pathways) and induced growth inhibition in human prostate cancer cells [57]. Given the oncogenic role of many of these client proteins (e.g., EGFR, SRC, and Raf-1), this specificity of Cdc37, rather than that of Hsp90, provides a potential and available therapeutic window for Cdc37-targeted therapy. The current studies of targeting Cdc37 were mainly focused on the application of the relevant gene silencers (e.g., shRNA/siRNA). With the development of microRNA for protein mediation [58], regulating microRNA targeting Cdc37 to decrease Cedc37 should also be taken into consideration.

#### Targeting Cdc37-client interaction

As mentioned above, phosphorylated Cdc37 at the conserved Ser13 site is a prerequisite for the efficient binding activity of Cdc37 to protein kinases and crucial for the recruitment of the protein kinase-Cdc37 complex to Hsp90 [47, 59, 60]. The phosphorylation of Cdc37 (Ser13) is mediated by CK2 [61, 62]. Specific inhibition of CK2 with its chemical inhibitor, 4,5,6,7-tetrabromobenzotriazole, could reduce the phosphorylation of Cdc37 and the protein levels of Cdc37-dependent protein kinases [59]. Besides the phosphorylation, the dephosphorylation of Cdc37 is pivotal for its activation as well. PP5, a serine/threonine-protein phosphatase that regulates hormone- and stress-induced cellular signaling [48, 63, 64], mediates Cdc37 dephosphorylation and is critically dependent for client protein release. Dephosphorylation is also important for the reversal of Cdc37’s resistance to nonspecific phosphatases and constitutive phosphorylation [48, 65]. These results show the regulatory mechanisms of Cdc37 activity and the molecular basis for the ability of CK2 and PP5 to regulate cellular functions of the Cdc37-client protein complex. Moreover, the finding that the phosphatase ability of PP5 is active only when it is interacting with proteins (e.g., Hsp90) via its TPR domain [66] has provided further evidence for targeting PP5 as a relatively specific means of blocking Hsp90 chaperone machinery.

Specific ATP-competitive kinase inhibitors may be another kind of candidates that block Cdc37 interaction with protein kinases. The kinase inhibitors (e.g., the B-raf inhibitor vemurafenib and the EGFR/HER2 inhibitor lapatinib) were found to antagonize Cdc37 interaction with target kinases not only by inducing the chaperone deprivation of nascent protein but also by disrupting the formed Hsp90-Cdc37-kinase complex. This Cdc37 antagonism finally leads to kinase depletion in a manner similar to that of Hsp90 inhibitor. Thus, the kinase inhibitors, besides the role that they are meant to play, also seem to act as antagonists of PPI within the Hsp90 molecular chaperone machinery [67]. These results also suggest that in some dependent tumor cells, Cdc37 chaperone deprivation, at least in part, contributes to the efficiency of targeted kinase inhibitors.

#### Targeting Hsp90-Cdc37 interaction

During the past few years, several kinds of disruptors toward the association of Hsp90 and Cdc37 have been discovered, as shown in Table 2. The underlying mechanisms of this kind of compounds mainly focus on the replacement of key residue–residue interaction between Hsp90 and Cdc37. Most of these disruptors are natural products, such as celastrol [68, 69], sulforaphane [70], FW-04-806 [71, 72], withaferin A [73–75], kongensin A [76, 77], and platycodin D [78, 79]. In addition to the small molecules, the peptide-derived disruptors also offer another way to block the interaction of Hsp90-Cdc37. For instance, the developed Cdc37-derived peptide Pep-1 exhibited competitive binding affinity to Hsp90 at the micromolar level though occupying the Cdc37 binding site. This binding subsequently leads to the disruption of Hsp90–Cdc37 PPI and the ATP bond to Hsp90 [80].

**Table 2:** Targeting the Hsp90-Cdc37-client protein interaction to disrupt Hsp90 chaperone machinery

The potential approaches to disrupt the Hsp90-Cdc37-client protein interaction	Related mechanisms	References
Targeting Cdc37		
siRNAs or shRNA	Silencing Cdc37	[56, 57]
Targeting Cdc37-client interaction		
4,5,6,7-Tetrabromobenzotriazole	CK2 inhibitors, suppressing Cdc37 phosphorylation at Ser13 site	[59]
PP5 mutation	Suppressing Cdc37 dephosphorylation	[48]
Vemurafenib	B-raf inhibitor. Antagonize Cdc37 interaction with kinases	[67]
Lapatinib	EGFR/HER2 inhibitor. Antagonize Cdc37 interaction with kinases	[67]
Targeting Hsp90-Cdc37 interaction		
Celastrol	Blocking the critical interaction of Hsp90 at Glu33 and Cdc37 at Arg167	[68, 69]
Sulforaphane	Disrupting the formation of Hsp90-Cdc37 complex by direct modification of specific amino acids residues of Hsp90	[70]
FW-04-804	Binding sites at Gln133 and Glu47/Arg46 of Hsp90	[71, 72]
Withaferin A	Blocking several H-bond between Hsp90-Cdc37 interaction, like Ser113 (Hsp90)-Gln208 (Cdc37) bond and Gln133 (Hsp90)-Arg166 (Cdc37) bond	[73–75]
Kongensin A	Covalently binds to a cysteine 420 in the middle domain of Hsp90 and dissociates Hsp90 from Cdc37	[76, 77]
Platycodin D	H-bond connection with Hsp90 at Arg32 and Phe200 and Cdc37 at Asp169 and Asp170	[78, 79]
Pep-1	Cdc37-derived peptides, bound to Hsp90 N-terminal domain and inhibited Hsp90 ATPase activity	[80]

There are three potential approaches to disrupt the function of Hsp90 chaperone machinery: targeting Cdc37, Cdc37-client protein interaction, and Hsp90-Cdc37 interaction

## Conclusion

The Hsp90 chaperone machinery is known to be a multi-protein complex, which includes the chaperone (Hsp90) and co-chaperones (e.g., HOP, Cdc37, and AHA1). This chaperone machinery has been regarded as prime importance to cancer survival with its effort on assistance of general protein folding and prevention of protein misfolded or unfolded actions. Hsp90, the famous heat shock protein, is the key factor for this chaperone machinery and allows cancer cells to adapt to severe stress [55, 81]. In the past decade, tremendous efforts have been made to develop Hsp90 inhibitors as anticancer agents [2, 82, 83]. However, none of the directed Hsp90 inhibitors have achieved the goal and have been applied in clinical treatment [2, 18, 19]. Seeking for another alternative strategy to inhibit the Hsp90 chaperone machinery and achieve better therapeutic efficiency is thus urgent.

Being the crucial recognizer of protein kinases to tie to the Hsp90 system, Cdc37 may be a promising candidate target. Most of the client proteins of Hsp90-Cdc37 are highly involved in key signal transduction systems and regulate cellular activities [84–86]. Besides the well-known client proteins of Hsp90-Cdc37 (e.g., HER2, CDK4, CDK6, and AKT), the key signaling molecules of necroptosis, the activation of receptor-interacting protein kinases 1–3 (RIP1–3), were found to be dependent on the interaction with the Hsp90-Cdc37 complex [76, 87, 88], which may help to generate a new approach against tumors with apoptotic deficiency. Androgen receptor (AR) is not a kinase protein but is recognized as a specific client protein of Hsp90-Cdc37 [89]. Given the key role of AR for transcriptional mediation in prostate cancer, the feasibility of targeting Hsp90–Cdc37 activity to mediate AR may be useful for prostate cancer treatment. Nevertheless, the resistance to directed Hsp90 inhibitors could also be reversed by silencing Cdc37 via destabilization of client proteins. This evidence supports that the blockage of Hsp90-Cdc37-client protein interaction can be an effective target for discovering novel inhibitors toward Hsp90 chaperone machinery and shows the potential to be applied in cancer treatment.

To date, several approaches are proven useful in blocking the association of Hsp90-Cdc37-client protein (as shown in Table 2); however, challenges remain. Firstly, the application of Cdc37-targeted intervention has not been fulfilled in vivo. To improve the stabilization and the transfection efficiency of RNA interference-Cdc37 [90] and to figure out the clear microRNA that targets Cdc37 will have superior therapeutic effect. Secondly, although the CK2 and PP5 are required for efficient phosphorylation and dephosphorylation of Cdc37, the CK2- or PP5-targeted therapies for clinical application are still under investigation [91]. Thirdly, the reported inhibitors of the Hsp90-Cdc37 complex so far are mainly natural products with poor selectivity, which may limit their potency to be drug candidates. The basic research of the Cdc37-derived peptides is still ongoing [92]. The structure modification of the current agents may provide further pharmacological properties of current agents while retaining target specificity of Hsp90-Cdc37 complex.

More still needs to be explored regarding the crucial role of Hsp90-Cdc37-client protein interaction and its effectiveness as target for cancer treatment. As mentioned above, depletion of Cdc37 may lead to the decrease of cell survival signals through either the degradation of the client protein level or the inhibition of client protein activity [56, 57]. The inhibition of Hsp90–Cdc37 interaction by the platycodin D also caused distinct regulation of client proteins as demonstrated by decreased activity and conserved protein level of AKT [78, 93]. Thus we remain uncertain of the full information of cancer types mediated by the regulation of Hsp90-Cdc37-client protein interaction. In addition, studies indicated the dysregulation of some client proteins obviously prefers certain cancer to other normal tissues. As AR is essential for the development of prostate cancer, and EGFR is a key factor for definite lung cancer, future research will be indispensable to pay attention to the stratification of patients before the application of Hsp90-Cdc37-client protein targeted therapy. Furthermore, it is not likely that agents that target Hsp90-Cdc37-client protein interaction will be used as mono-therapy [56, 72, 78]. Rather, the known biological role and pre-clinical findings demonstrate that regulation of this complex will have more potential in the modulation of therapeutic response of other agents in certain cancers.

In conclusion, targeting Hsp90-Cdc37-client protein interaction is a reasonable and alternative strategy to the blockage of Hsp90 chaperone machinery and is a promising target for the cancer therapy.

## Additional file

Additional file 1: The sup. Excel file for the comprehensive analysis of Hsp90-Cdc37’s client proteins. Comprehensive Analysis of Hsp90-Cdc37’s client proteins by using FunRich tool. (XLSX 313 kb)

## Abbreviations

AHA1: Activator of Hsp90 ATPase protein 1
AR: Androgen receptor
Cdc37: Cell division cycle 37
CeCdc37: Cdc37 within *Caenorhabditis elegans*
CHIP: Carboxyl terminus of HSC70-interacting protein
CK2: Casein kinase 2
CYP40: Cyclophilin 40
FKBP5: FK506-binding protein 5
GM: Geldanamycin
HOP: Hsp70/Hsp90 organizing protein
Hsp90: Heat shock protein 90
PP5: Protein phosphatase 5
PPI: Protein–protein interaction
RIP1–3: Receptor-interacting protein kinases 1–3
SGTA: Small glutamine-rich TPR-containing protein alpha
S-TKc: Serine/threonine kinase catalytic domain
TAH1: TPR repeat-containing protein associated with Hsp90
TTC4: Tetratricopeptide repeat domain 4
Unc45: Uncoordinated mutant number 45
